# A sonographic software program, Fluctuational Imaging, for diagnosis of hepatic hemangioma

**DOI:** 10.1038/s41598-022-08482-9

**Published:** 2022-03-18

**Authors:** Hiroshi Imamura, Jiro Hata

**Affiliations:** Division of Endoscopy and Ultrasound, Department of Clinical Pathology and Laboratory Medicine, Kawasaki Medical School, 577 Matsushima, Kurashiki, Okayama 701-0192 Japan

**Keywords:** Gastroenterology, Software

## Abstract

Hepatic hemangioma is the most common benign solid lesion of the liver. Contrast-enhanced computed tomography or magnetic resonance imaging is recommended for definitive diagnosis of hepatic hemangioma. However, these modalities have drawbacks in terms of radiation exposure, invasiveness, and high cost for examination. “Fluttering sign” is one of the candidate findings considered specific for hepatic hemangioma that can be useful for diagnosis of hepatic hemangioma using grayscale US alone. However, the assessment is subjective and the findings are weak and likely to be overlooked in some cases. We developed a software program, Fluctuational Imaging, for objective detection and depiction of “fluttering sign”. Here, we evaluated the ability of Fluctuational Imaging software to depict “fluttering sign” in hepatic hemangioma. Presence or absence of “fluttering sign” was evaluated in the grayscale US videos and Fluctuational Imaging software analysis results of patients with hepatic hemangioma. The Cohen’s kappa test showed very good agreement (0.95). Fluctuational Imaging software can detect and depict the phenomenon of “fluttering sign” well and may be a useful tool for diagnosis of hepatic hemangioma.

## Introduction

Hepatic hemangioma is the most common solid lesion of the liver with an incidence ranging from 1 to 20% in the total population^1–3^. Hepatic hemangioma consists of blood-filled vascular spaces of different sizes lined by a simple layer of endothelial cells and supported by a fibrous stroma^1–3^, and is actually a venous malformation rather than a hemangioma^4^.

The typical hepatic hemangioma appears as a hyperechoic homogeneous nodule with well-defined margins and posterior acoustic enhancement on ultrasonography (US)^1–3^. However, other lesions often present similar US appearances to hepatic hemangioma^5^, and the ability to differentiate hepatic hemangioma from other hepatic lesions using grayscale US and Doppler US techniques is limited^6^. For definitive diagnosis of hepatic hemangioma, performance of contrast-enhanced US, contrast-enhanced computed tomography, or magnetic resonance imaging is recommended^7^. However, these modalities have drawbacks in terms of radiation exposure, invasiveness, high cost for examination, and risk of allergic reaction to contrast agents, given that hepatic hemangioma has a high incidence and is a benign lesion.

There are few reports on candidate findings considered specific for hepatic hemangioma that can be useful for diagnosis of hepatic hemangioma using grayscale US alone^8–15^. “Fluttering sign”^14^ and “tubifex-like fine echo movement”^15^, thought to reflect the same phenomenon, are phenomena wherein the internal echogenicity of hepatic hemangioma changes continuously during US examination and seems to be moving. The speculated mechanisms for “fluttering sign” are deformation of the internal structure of the hepatic hemangioma through arterial pulsation, slow blood flow in the hepatic hemangioma, and acoustic streaming of the blood flow in the hepatic hemangioma. The phenomenon may be useful for diagnosis, but is a subjective assessment. Considering that “fluttering sign” can be evaluated within a few seconds and that objective investigation of changes in signal strength over time should be possible using imaging technology, we developed a software program named Fluctuational Imaging for objective detection and depiction of “fluttering sign”.

The purpose of this study was to evaluate the ability of Fluctuational Imaging software to detect and depict “fluttering sign”.

## Methods

This study involving retrospective interpretation of prospectively acquired data was approved by the Kawasaki Medical School ethics committee. The study was performed in accordance with the Declaration of Helsinki. Informed consent was obtained from all participants.

### US examinations

The US equipment used was a TUS-AI900 system with 4-MHz convex or 7-MHz linear probes (Canon Medical Systems Corporation). Patients were placed in the supine position and asked to hold their breath, rather than performing the Valsalva maneuver, while the grayscale US video was acquired. The focus of the US beam was set at the lowest (deepest) margin of the lesion, and software that may influence the results of Fluctuational Imaging software analysis, such as enhancement of structure boundaries or multidirectional beaming, was turned off during data acquisition. The grayscale US video of each lesion was recorded for about 5 s as clip data on an inbuilt hard disk. All US examinations were performed by a single doctor who was an expert in abdominal US (35 years of experience).

### Patients

Grayscale US videos of 76 patients with hepatic hemangioma were saved from June 2018 through May 2019. The eligibility criteria were as follows: age ≥ 18 years, ability to hold their breath, and potential for acquisition of a clear grayscale US video. Finally, we enrolled 62 patients who had confirmed diagnoses and could be analyzed with Fluctuational Imaging software. Diagnosis of hepatic hemangioma was confirmed by contrast-enhanced US (21 patients), contrast-enhanced computed tomography (17 patients), or magnetic resonance imaging (4 patients), or by typical appearance on US and absence of changes in morphology and size of the lesion for more than 1 year (20 patients).

### Fluctuational imaging software

Fluctuational Imaging is a software program for detection and depiction of “fluttering sign” in grayscale images. The software was developed with consideration of the lesion type using the program language of Visual C +  + 2010. Because “fluttering sign” is a phenomenon in which the signal intensity changes on the time axis, a cross-correlation coefficient was evaluated. The frame rate was 20 frames/s and 70 frames (3.5 s) were used for Fluctuational Imaging software analysis. The time required for Fluctuational Imaging software analysis was approximately 10–20 s. The procedure is shown below.

1. Manual determination of the region of interest according to the size of the lesion
2. Elimination of motion effects of the heart or arterial pulsation using “motion tracking” technology that can track and cancel overall movement in the region of interest to reduce the effects of body movements arising from pulsation and breathing
3. Calculation of “R”, a cross-correlation coefficient, for each point in the region of interest using data within a range of few millimeters (approximately 2 mm)
  $$R = \frac{{\mathop \sum \nolimits_{i} \mathop \sum \nolimits_{j} \left( {I_{n} \left( {i,j} \right) - \overline{I}_{n} } \right)\left( {I_{n - 1} \left( {i,j} \right) - \overline{I}_{n - 1} } \right)}}{{\sqrt {\mathop \sum \nolimits_{i} \mathop \sum \nolimits_{j} \left( {I_{n} \left( {i,j} \right) - \overline{I}_{n} } \right)^{2} } \sqrt {\mathop \sum \nolimits_{i} \mathop \sum \nolimits_{j} \left( {I_{n - 1} \left( {i,j} \right) - \overline{I}_{n - 1} } \right)^{2} } }}$$
  $\overline{I}$: luminosity value; n: frame number; i, j: coordinates of the point.
4. Calculation of degree of coincidence of the cross-correlation coefficient around the analysis point.
  Degree of fluctuation: change in local signal strength along the time axis.
  Large degree of fluctuation: degree of coincidence was small.
5. Assignment of “R” 0–1 to 255 gradation colors
  Large degree of fluctuation: yellow to red.
  Small degree of fluctuation: blue.

Because the analysis was qualitative rather than quantitative, there was no specific cutoff value for “R” and no degree of coincidence value above or below which a specific classification of the lesion was made. Procedure numbers 2 to 4 interacted for analysis. Based on the results of preliminary research, 255 gradation colors were assigned to emphasize “fluttering sign”.

Canon Medical Systems Corporation has the following patent: Y. Honjo et al., Analyzing Apparatus and Analyzing Program, P2020-54815A, 2020-04-09.

The procedure for Fluctuation Imaging software analysis.

1. Save a grayscale US video for about 3–5 s
2. Launch the Fluctuation Imaging software built into the US equipment
3. Manually determine the region of interest
4. Determine the number of frames used for analysis
5. Perform the Fluctuational Imaging software analysis..

### Evaluation of “fluttering sign” in grayscale US videos

An experienced medical doctor (12 years of experience) repeatedly reviewed playback of the grayscale US videos without patient information and judged the presence or absence of “fluttering sign” subjectively. Presence of “fluttering sign” was defined as continuous changes in echogenicity observed in the hepatic hemangioma.

### Evaluation of Fluctuational Imaging software analysis results

A positive Fluctuational Imaging software analysis result was defined as presence of colored areas such as yellow or red in the hepatic hemangioma.

### Agreement between Fluctuational Imaging software and grayscale US video results

The agreement between Fluctuational Imaging software analysis results and grayscale US video results regarding the presence or absence of “fluttering sign” was evaluated by Cohen’s kappa coefficient. Statistical analysis was performed with EZR (Jichi Medical University, Japan), which is a graphical user interface for R^16^.

## Results

### Patient demographics

A total of 62 patients were enrolled. The patients comprised 34 women and 28 men (mean age ± SD: 55.8 ± 13.7 years; range 27–80 years).

### “Fluttering sign” in grayscale US videos

“Fluttering sign” was observed in 34 hepatic hemangiomas, giving an appearance rate of 54.8% (34 of 62). Supplementary video V1 shows a grayscale US video with noticeable “fluttering sign”, Supplementary video V2 shows a grayscale US video without “fluttering sign”, and Supplementary video V3 shows a grayscale US video with weak “fluttering sign”.

### Fluctuational Imaging software analysis results

**Figure 1:**
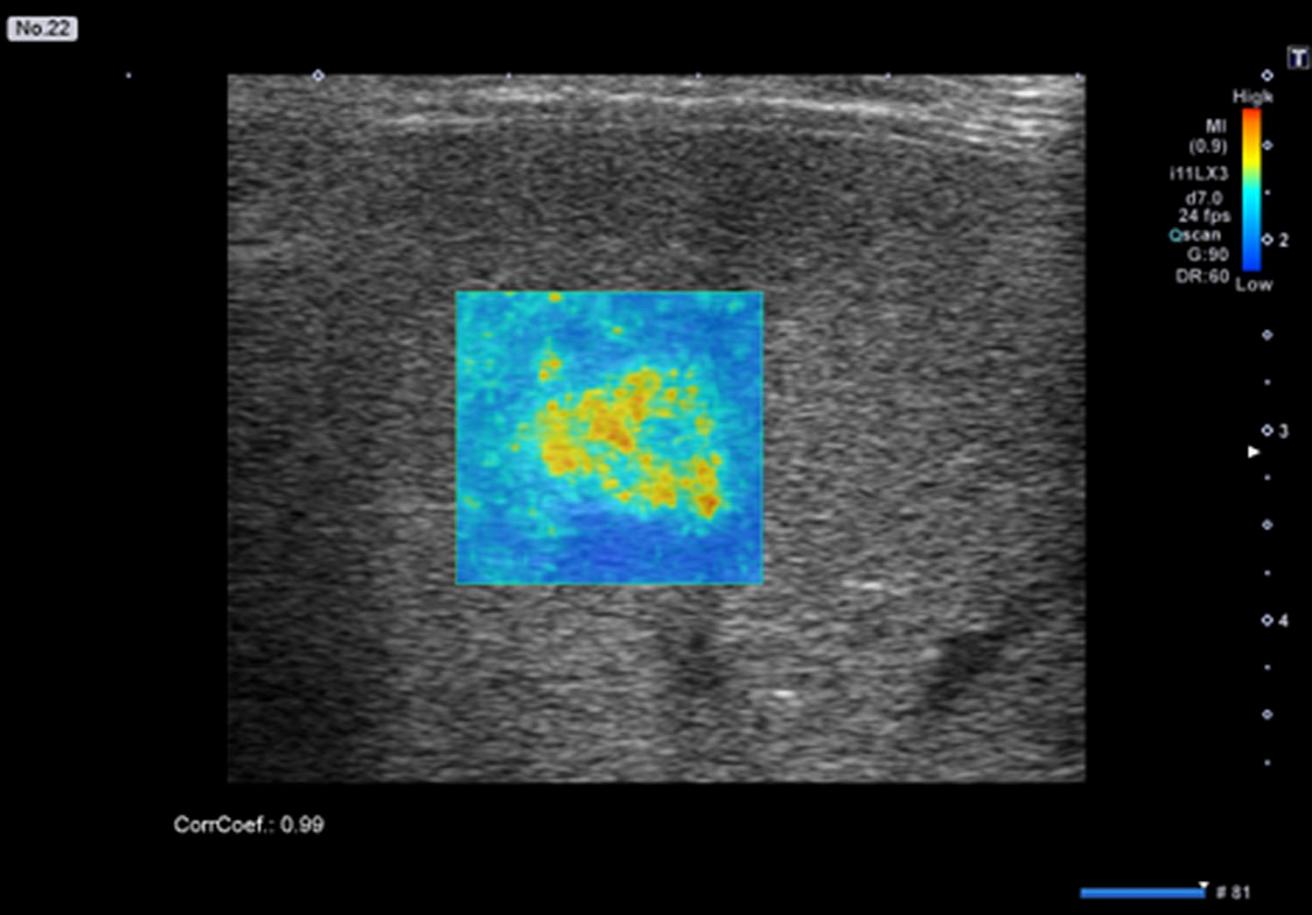
Fluctuational Imaging analysis results for a hepatic hemangioma with a noticeable “fluttering sign”. The analysis shows a colored area in the nodule.

**Figure 2:**
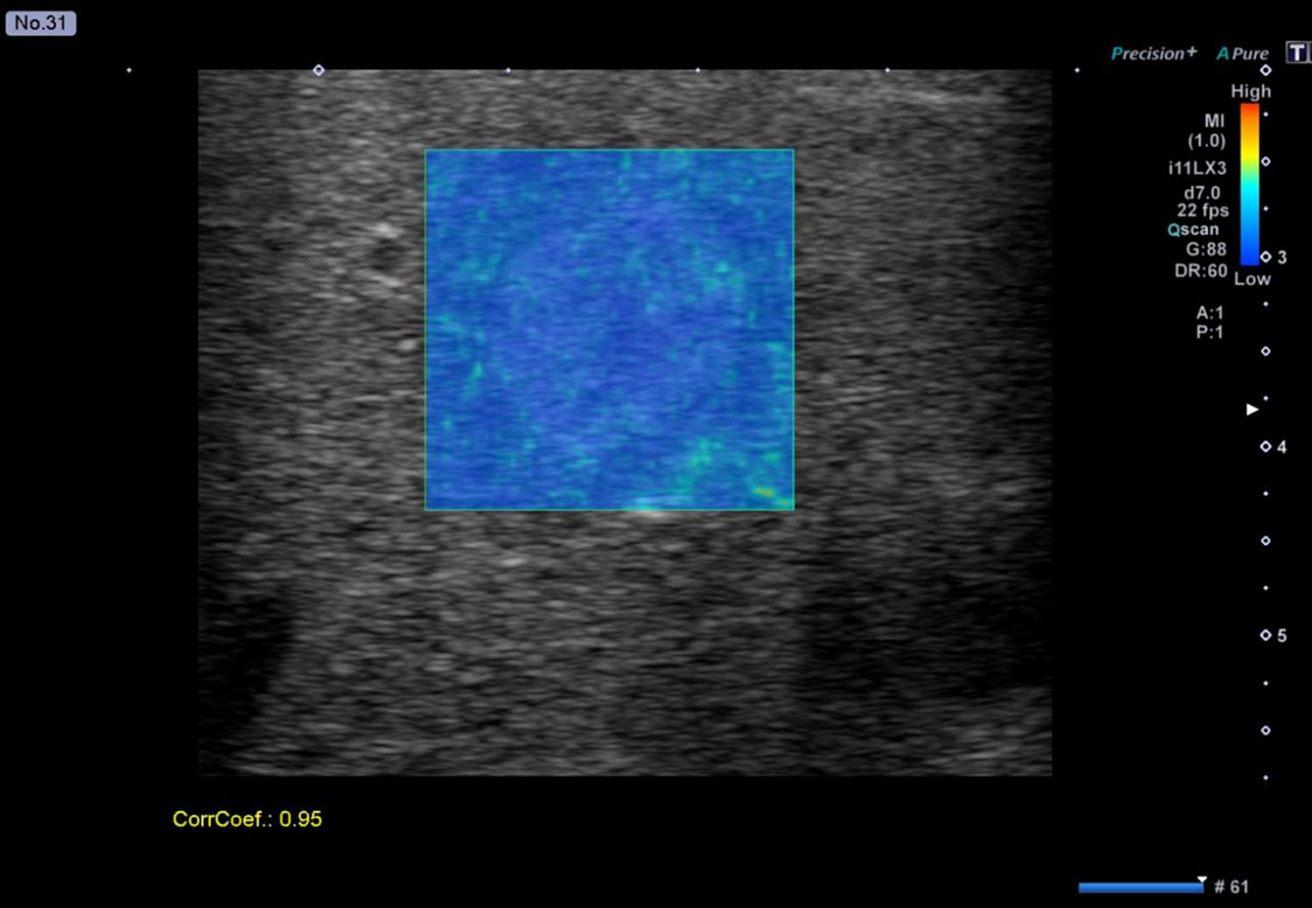
Fluctuational Imaging analysis results for a hepatic hemangioma without “fluttering sign”. The analysis shows no colored area in the nodule.

**Figure 3:**
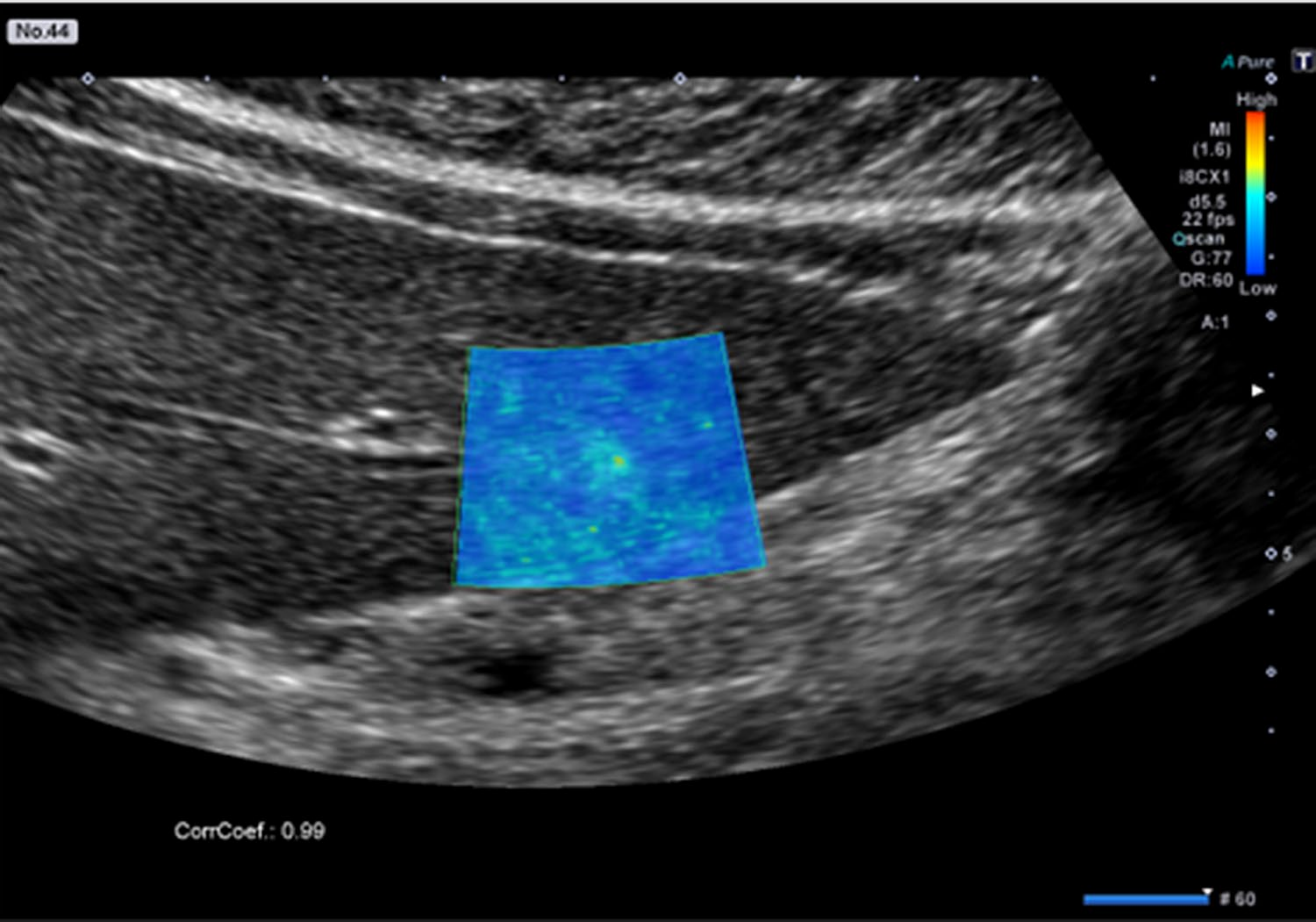
Fluctuational Imaging analysis results for a hepatic hemangioma with a weak “fluttering sign”. The analysis shows a colored area in the nodule.

Fluctuational Imaging software analysis results were positive for “fluttering sign” in 38 hepatic hemangiomas. Figures 1, 2 and 3 shows the results for Supplementary video V1, Supplementary video V2, and Supplementary video V3, respectively.

Agreement between Fluctuational Imaging software analysis results and grayscale US video results.

Table 1 shows Fluctuational Imaging software analysis results and grayscale US video results.

**Table 1:** The findings of the grayscale ultrasonography and Fluctuational Imaging software analysis results.

Fluctuational Imaging software	Grayscale US video	
	Positive	Negative
Positive	34	4
Negative	0	28

The Cohen’s kappa test between the Fluctuational Imaging software analysis results and the grayscale US video results regarding the presence or absence of “fluttering sign” showed very good agreement (0.95 [0.74–0.99]).

**Figure 4:**
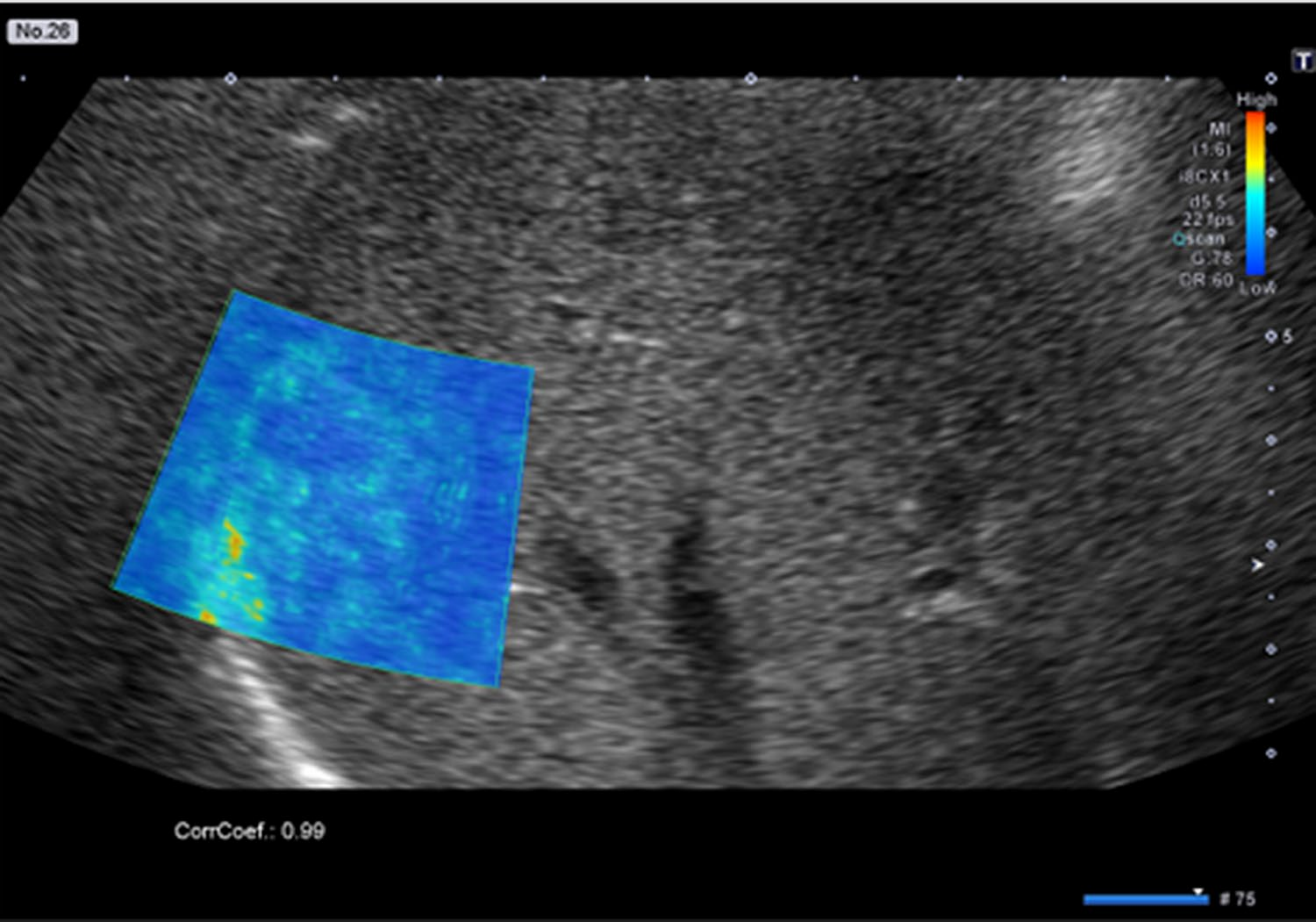
Fluctuational Imaging analysis results for a hepatic hemangioma considered to be false-positive on Fluctuational Imaging software analysis because of the effect of pulsation. The analysis shows a colored area in part of the region contacting the diaphragm.

Four cases with different results in the grayscale US videos and Fluctuational Imaging software analysis were negative on grayscale US videos and positive on Fluctuational Imaging software analysis. The different results were considered to arise from the effects of pulsation. Figure 4 shows a colored area thought to arise from an effect of the heartbeat rather than “fluttering sign” (Supplementary video V4).

## Discussion

This initial and preliminary report demonstrates that the sonographic software program Fluctuational Imaging was able to detect and depict “fluttering sign” in hepatic hemangioma.

“Fluttering sign” is considered a specific finding for hepatic hemangiomas and its appearance rate is about 50–70%^14,15^. The appearance rate of “fluttering sign” in the present study was similar. Even though “fluttering sign” is only observed in about 50–70% of hepatic hemangiomas, it can be a useful finding for diagnosis of hepatic hemangioma using grayscale US videos alone when it is present. Similarly, Fluctuation Imaging software is only useful for cases in which “fluttering sign” is observed.

Because there is no standard for judging the presence or absence of “fluttering sign”, we determined its presence or absence by repeatedly reviewing playback of the grayscale US videos in the present study. We then evaluated the performance of the Fluctuational Imaging software analysis based on the degree of matching with the grayscale US video results. The fact that the grayscale US video and Fluctuational Imaging software analysis results were almost the same does not mean that Fluctuational Imaging software analysis is unnecessary, because repeated playback of grayscale US videos is not possible during actual US examinations. There were cases that demonstrated the usefulness of Fluctuational Imaging software analysis results because the “fluttering sign” was weak and likely to be overlooked. Meanwhile, a few cases were considered to be false-positives for Fluctuational Imaging software analysis, because the internal echogenicity of the hepatic hemangioma did not change continuously or seem to be moving. It may be better to use Fluctuational Imaging software for areas without continuous motion, because the effect of pulsation was considered the cause of the false-positive cases. Further studies are warranted to determine how to optimize Fluctuational Imaging software analysis.

As hepatic hemangiomas are common benign lesions and biopsy involves a risk of bleeding, the diagnosis of hepatic hemangioma was made based on a previous report^17^ and guidelines^7,18^. There are several subtypes of hepatic hemangioma, including cavernous hemangioma and capillary hemangioma. Because the differences in “fluttering sign” and Fluctuational Imaging software analysis for subtypes of hepatic hemangioma were not evaluated, further studies are required to validate the software analysis for the subtypes of hepatic hemangioma.

Because a saved grayscale US video is analyzed, Fluctuational Imaging software is not invasive and has no associated cost, but real-time evaluation is not possible. The analysis using Fluctuational Imaging software is easy and the time required is about 1–2 min. The analysis requires surrounding tissues of approximately the same size as the hepatic hemangioma for comparison. Therefore, the analysis is theoretically considered possible for a hepatic hemangioma in which an ROI of twice its area can be set. However, the size criteria for a hepatic hemangioma for use of Fluctuational Imaging software analysis have not been determined, and further studies are needed. Two different transducers were used in the present study, but the effect of the differing frequencies was not examined. Although the spatial resolutions of the images collected with the 4-MHz convex or 7-MHz linear transducers were different, the same algorithm was used, and thus it is possible that the colored parts differed slightly between the two transducers. Evaluation of the effect of the different transducers is necessary.

Because this is an initial and preliminary report involving a small number of cases, the study has several limitations. Specifically, because the statistical agreement was based on the observations of only one reviewer, the inter-rater or intra-rater agreement was not assessed, some biases may be present, and the reproducibility was not assessed. Studies with larger numbers of hepatic lesions are needed to evaluate the reproducibility, the effect of lesion position in the liver, the differential diagnosis ability for focal liver lesions, and the clinical usefulness of Fluctuational Imaging software.

In conclusion, we have developed a software program named Fluctuational Imaging. Fluctuational Imaging software can detect and depict the phenomenon of “fluttering sign” well and may be a useful tool for diagnosis of hepatic hemangioma.

## Supplementary Information

Supplementary Legends.

Supplementary Video 1.

Supplementary Video 2.

Supplementary Video 3.

Supplementary Video 4.

## Data Availability

The datasets generated during and/or analyzed during the current study are available from the corresponding author on reasonable request.
