# Supplementary material for: A sonographic software program, Fluctuational Imaging, for diagnosis of hepatic hemangioma
Source: Sci Rep. 2022 Mar 18;12:4701. doi: 10.1038/s41598-022-08482-9 (PMC8933546; doi:10.1038/s41598-022-08482-9)
Supplement: Supplementary file 1 — Supplementary Legends. [file 41598_2022_8482_MOESM1_ESM.pdf]

A sonographic software program, Fluctuational Imaging, for diagnosis of hepatic hemangioma

*Hiroshi Imamura, Jiro Hata

Supplementary video V1
Title: Grayscale US movie of hepatic hemangioma with noticeable “fluttering sign”.
Legend: The video shows “fluttering sign” in the nodule.

Supplementary video V2
Title: Grayscale US movie of hepatic hemangioma without “fluttering sign”.
Legend: The video does not show “fluttering sign” in the nodule.

Supplementary video V3
Title: Grayscale US movie of hepatic hemangioma with weak “fluttering sign”.
Legend: The video shows “fluttering sign” in the upper right part of the nodule.

Supplementary video V4
Title: Grayscale US movie of hepatic hemangioma considered to be false-positive for Fluctuational Imaging software due to the effect of pulsation.
Legend: The video does not show “fluttering sign” in the nodule.
